# Regulation of the immune microenvironment and immunotherapy after liver transplantation

**DOI:** 10.3389/fimmu.2025.1602877

**Published:** 2025-05-12

**Authors:** Tianyi Lan, Gang Wu, Bangyou Zuo, Jingming Yang, Pan He, Yu Zhang

**Affiliations:** ^1^ Department of Hepatobiliary and Pancreatic Surgery, Sichuan Provincial People’s Hospital, University of Electronic Science and Technology of China, Chengdu, China; ^2^ School of Medicine, University of Electronic Science and Technology of China, Chengdu, China; ^3^ Southwest Medical University, Luzhou, Sichuan, China

**Keywords:** end-stage liver disease, liver transplantation, immune microenvironment, immune regulation, immunotherapy

## Abstract

Liver transplantation (LT) is a primary treatment option for patients with end-stage liver disease. However, post-transplantation immune regulation is critical to graft survival and long-term patient outcomes. Following liver transplantation, the recipient’s immune system mounts a response against the graft, while the graft promotes anti-rejection immune reactions and the establishment of immune tolerance. In recent years, advances in the study of the immune microenvironment have provided new insights into post-transplantation immune regulation. Meanwhile, immunotherapy strategies have opened new possibilities for improving transplantation success rates and long-term survival. This review summarizes recent progress in understanding the immune microenvironment and immunotherapy following liver transplantation, focusing on key components of the transplant immune microenvironment, their regulatory networks and mechanisms, major immunosuppressive strategies, emerging immunotherapeutic approaches, and current challenges. The aim was to provide a theoretical foundation for optimizing clinical practice.

## 1 Introduction

**Figure 1:**
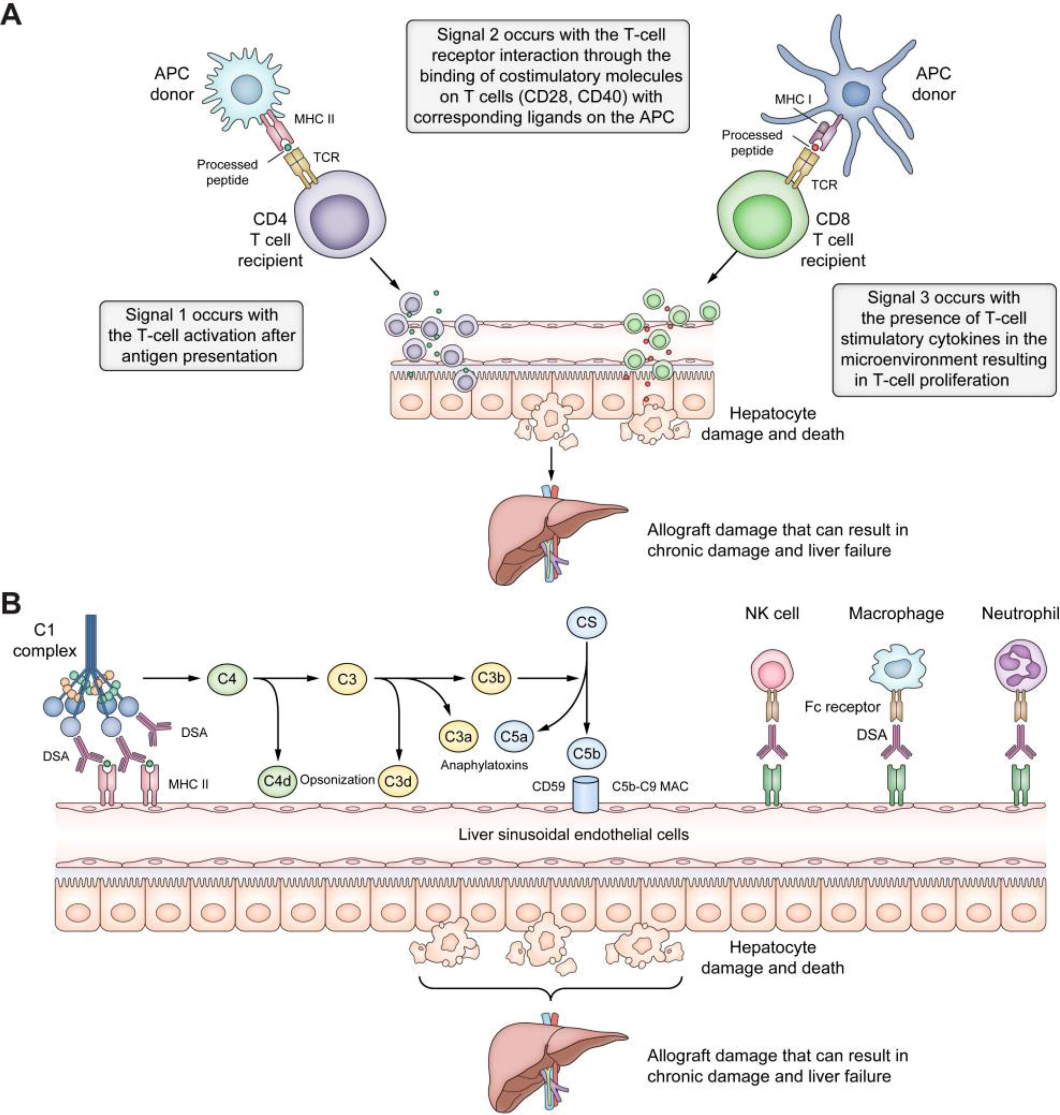
Pathways of graft rejection or damage after liver transplantation. **(A)** T-cell allorecognition pathways after liver transplantation. In the direct pathway, the host’s T-cell receptors interact with the MHC class I and II antigens on the donor’s APCs. In the indirect pathway, MHC antigens are internalized by the recipient’s APCs, processed, and presented as peptide fragments to the host’s CD4^+^ and CD8^+^ T cells. The semi-direct pathway entails membrane trafficking between donor and host cells or extracellular vesicles. Effective allorecognition requires three signals: Signal 1, T-cell activation after antigen presentation; Signal 2, T-cell-receptor interaction through the binding of costimulatory molecules on T cells (CD 28, CD40) with the respective ligands on the APC (CD40L, CD80, CD86); Signal 3, T-cell stimulation by cytokines in the microenvironment resulting in T-cell proliferation and damage to the liver allograft. **(B)** Antibody-mediated rejection pathways. Antibody-mediated rejection can occur after the DSAs bind to MHC antigens on the liver allograft, leading to classical complement pathway activation by binding to the C1 complex. The complement system can damage the liver allograft through a) opsonization, where C4d and C3d bind to liver cells, tagging them for elimination by the innate immune system; b) anaphylatoxin, where C3a and C5a act as strong chemotactic signals, engaging inflammatory cells and leading to tissue damage; c) the MAC, where C5b-9 MAC can injure cells by perforating holes in the cell membranes. Additionally, by engagement with the FC receptor, DSAs can bind MHC molecules to promote the recruitment of innate immune system cells, such as neutrophils, macrophages, and NK cells. APCs, antigen-presenting cells; DSAs, donor-specific antibodies; MAC, membrane attack complex; MHC, major histocompatibility complex; NK natural killer (4). Adapted with permission from copyright 2023, Elsevier Ltd.

Since its first successful implementation in the 1960s, liver transplantation (LT) has become the optimal treatment for end-stage liver disease, driven by decades of technological advancements and improvements in perioperative management (1). The long-term functional stability of the transplanted liver relies on precise regulation of the immune system to prevent rejection. Optimizing immunosuppressive strategies and developing individualized treatment regimens can significantly reduce the risk of acute and chronic rejection, improving graft survival and patient outcomes (2, 3). It was found that cell-mediated acute rejection and donor-specific antibody-induced chronic rejection after LT can lead to progressive fibrosis and bile duct injury in the transplanted liver, affecting graft function. The pathways involved in graft rejection or injury after transplantation are shown in **Figure 1** (4, 5). However, excessive immunosuppression is associated with severe clinical complications, including nephrotoxicity, hypertension, diabetes, osteoporosis, increased infection risk, and a higher incidence of malignancies (6). Long-term use of calcineurin inhibitors (such as tacrolimus and cyclosporine) has been significantly associated with chronic renal dysfunction, while glucocorticoid therapy may exacerbate metabolic syndrome and osteoporosis progression (7). Such adverse effects reduce the patient’s quality of life and significantly shorten their survival.

A key challenge in post-transplantation immunotherapy is achieving a balance between minimizing immunosuppression-related adverse effects and preventing structural and functional damage to the graft. This requires a comprehensive understanding of the dynamic interactions between innate and adaptive immune cells in the post-transplantation microenvironment, as well as the integration of biomarkers to guide personalized immunosuppressive regimens. This review summarizes recent advances in understanding the immune microenvironment and immunotherapy after LT, focusing on key components of the transplant immune microenvironment, their regulatory networks and mechanisms, current immunosuppressive strategies, emerging immunotherapeutic approaches, and existing challenges. The goal was to provide a theoretical foundation for optimizing clinical practice.

## 2 Post-transplantation immune microenvironment and its regulatory networks and mechanisms

The liver possesses inherent immune tolerance due to its unique anatomical structure and physiological functions. This tolerance primarily stems from its dual blood supply system: the portal vein and the hepatic artery. The portal vein accounts for approximately 80% of the liver’s total blood flow, delivering blood rich in nutrients, metabolic byproducts, and a high load of microbial antigens and endotoxins from the gastrointestinal tract, spleen, and pancreas. While the liver tolerates these immunological challenges, it must simultaneously maintain immune surveillance against pathogenic infections and tumor cells—a balance achieved through its distinctive immune microenvironment (8). After LT, donor-derived lymphocytes and dendritic cells (DCs) migrate to the recipient’s secondary lymphoid organs (such as lymph nodes and the thymus), where they release soluble major histocompatibility complex (MHC) molecules. This process induces the depletion and elimination of alloreactive T cells, thereby promoting immune tolerance (9). Additionally, liver sinusoidal endothelial cells (LSECs), characterized by their large intercellular gaps and unique phenotypic features, play a crucial role in immune tolerance. LSECs suppress T cell activation and induce apoptosis by expressing immunoregulatory molecules such as programmed death-ligand 1 (PD-L1) (10). Moreover, Kupffer cells (KCs), the resident macrophages of the liver, contribute to immune tolerance by phagocytosing antigen-antibody complexes and secreting anti-inflammatory cytokines such as interleukin-10 (IL-10) and transforming growth factor-beta (TGF-β). This immunosuppressive activity dampens immune responses, helping the graft resist attacks from the recipient’s immune system (11). The key immune cells and their regulatory functions in the post-transplantation immune microenvironment are discussed in detail below.

### 2.1 T and B cells in post-transplantation immunity

T and B cells are central to immune regulation, with their functional diversity and interactions critical for maintaining immune balance. T cells are classified into various subsets based on surface markers and functional characteristics, including cytotoxic T cells (CD8^+^ T cells) and helper T cells (CD4^+^ T cells) (12). CD4^+^ T cells can further differentiate into multiple subsets, such as Th0, Th1, Th2, Th3, and Th17 cells (13). Th1 cells secrete pro-inflammatory cytokines like IL-2 and interferon-gamma (IFN-γ), playing a key role in acute rejection, whereas Th2 cells predominantly produce anti-inflammatory cytokines such as IL-4 and IL-10, which are crucial for inducing immune tolerance and suppressing excessive immune responses (14). The dynamic balance between cytokines produced by Th1 and Th2 cells is a key mechanism in immune tolerance regulation. Studies have shown that in the early post-transplantation period, the number of Th1 cells significantly decreases while that of Th2 cells gradually increases, leading to a shift toward Th2 dominance. This immunophenotypic transition promotes immune tolerance and reduces the risk of acute rejection by increasing the secretion of anti-inflammatory cytokines (e.g., IL-4 and IL-10) while decreasing the production of pro-inflammatory cytokines (e.g., IL-2 and IFN-γ) (15).

The interaction between KCs and Th17 cells plays a crucial role in acute immune rejection following allogeneic LT (16). As resident liver macrophages, KCs promote the differentiation of naïve CD4^+^ T cells into Th17 cells rather than regulatory T cells (Treg cells) by secreting IL-6 and TGF-β (17). The combined action of IL-6 and TGF-β significantly enhances Th17 cell differentiation, and these further exacerbate inflammation and tissue damage by secreting pro-inflammatory cytokines such as IL-17 and IL-22, thereby facilitating acute rejection (18). Additionally, an imbalance between Th17 and Treg cells is considered a key mechanism underlying graft immune homeostasis disruption (19). B cells also play a significant role in liver transplant rejection. Studies suggest that B cell-mediated humoral immune responses are particularly prominent in chronic rejection and graft dysfunction (20).

### 2.2 Dendritic cells in post-transplantation immunity

DCs typically exhibit an immature phenotype, characterized by low expression of MHC class II (MHC-II) molecules, costimulatory molecules (such as CD80 and CD86), and the pro-inflammatory cytokine IL-12p70 while displaying high levels of immunoregulatory cytokines such as IL-10, IL-27, and TGF-β (21). This phenotype enables immature DCs to promote the proliferation of Tregs while inhibiting the activation of effector T cells, thereby facilitating immune tolerance after LT (22). Animal studies have demonstrated that immature DCs overexpressing IL-10 and the Fas ligand can significantly enhance immune tolerance by maintaining low expression levels of MHC-II and costimulatory molecules (23).

### 2.3 Kupffer cells in post-transplantation immunity

Non-parenchymal liver cells other than DCs, such as KCs and LSECs, also play critical roles. KCs perform essential physiological functions, primarily involving the efficient recognition and clearance of circulating pathogens and apoptotic cells. Incomplete clearance of apoptotic cells results in the accumulation of apoptotic debris, which can trigger inflammatory cascades. Consequently, the phagocytic capacity of KCs after transplantation is critical to maintaining immune tolerance (24). Following phagocytosis, KCs secrete multiple immunosuppressive cytokines, including TGF-β, IL-10, and prostaglandin E2. Through their synergistic effects, these cytokines establish an immunosuppressive microenvironment that negatively regulates lymphocyte function, inducing antigen-specific immune tolerance toward allografts (25).

### 2.4 Liver sinusoidal endothelial cells in post-transplantation immunity

LSECs mediate antigen uptake, processing, and MHC-I presentation through their specifically expressed scavenger receptor family members (represented by mannose receptors), driving immune tolerance responses in naïve CD8^+^ T cells. The molecular mechanism primarily involves upregulation of the immune checkpoint molecule PD-L1 on LSECs, which regulates T cell activation through the PD-1/PD-L1 co-inhibitory signaling pathway (10).

LSECs also express MHC-II molecules and possess the immunological function of antigen presentation to CD4^+^ T cells. However, due to their characteristic low expression of co-stimulatory molecules, LSECs cannot induce naïve CD4^+^ T cells to differentiate into helper T cell subsets effectively. Instead, they preferentially promote the generation and expansion of Tregs. This immunological property of LSECs induces immune tolerance by suppressing autoimmunity and inhibiting the release of inflammatory factors (26).

## 3 Current status of post-transplantation immunotherapy strategies

Immune rejection following LT is a primary factor affecting long-term graft survival. Treatment strategies must be individually tailored according to the type and severity of the rejection. The core objective of immunosuppressive therapy is to prevent the onset of rejection reactions and avoid graft damage mediated by irreversible changes in the immune microenvironment.

### 3.1 Common immunotherapy strategies after liver transplantation

Immune checkpoint inhibitors (ICIs), widely used in cancer immunotherapy, work by blocking immune checkpoint pathways such as PD-1/PD-L1 and CTLA-4, thereby enhancing T lymphocyte-mediated cytotoxicity against tumor cells and restoring antitumor immunity (27). However, their application after LT remains a significant challenge. By boosting T cell function, ICIs might disrupt the established immune tolerance of the graft, increasing the risk of acute or chronic rejection. Furthermore, ICIs can induce immune-related adverse events, further complicating post-transplantation management. Currently, the primary strategies to treat rejection after LT depend on the rejection type and severity, and include: 1) Mild T cell-mediated rejection (TCMR) is managed by increasing calcineurin inhibitors (CNIs). 2) Moderate to moderately severe TCMR is treated by intravenous pulse steroids and CNI therapy, followed by a slow taper of oral steroids. 3) Severe TCMR with significant graft damage and cholestasis is managed with antibody-depleting therapy as a first-line treatment. 4) Steroid-resistant TCMR is managed with antibody-depleting therapy such as anti-thymocyte globulin. The current status of these treatment approaches is discussed in the following sections.

### 3.2 Current status of immunotherapy after liver transplantation

In the early stages of organ transplantation, corticosteroids and azathioprine were the primary immunosuppressive agents used to modulate the recipient’s immune response and prevent graft rejection. However, prolonged use of these drugs led to immune dysregulation, graft dysfunction, and decreased patient survival rates (28). CNIs, such as cyclosporine and tacrolimus, revolutionized transplant immunosuppression by significantly improving graft survival and long-term patient outcomes (29). The mechanisms of action of CNIs include: 1) Competitive binding to the active site of calcineurin, preventing calcium-dependent activation and consequently inhibiting its enzymatic activity. This blockade disrupts intracellular signaling pathways crucial for T cell activation. 2) Selective inhibition of the transcription of IL-2 and other cytokines in T lymphocytes. Cyclosporine binds to cyclophilin, while tacrolimus binds to FK-binding protein, forming drug-receptor complexes that specifically inhibit calcineurin. This inhibition prevents nuclear translocation of NF-AT family transcription factors, thereby reducing the transcriptional activation of cytokines such as IL-2, TNF-α, IL-3, IL-4, CD40L, granulocyte-macrophage colony-stimulating factor, and IFN-γ, ultimately suppressing T cell proliferation and immune responses (30). Despite its effectiveness, tacrolimus has significant adverse effects, including neurotoxicity, nephrotoxicity, and an increased risk of tumor recurrence, severely impacting patients’ quality of life (31). Additionally, tacrolimus has a narrow therapeutic window and exhibits substantial pharmacokinetic variability influenced by genetic polymorphisms, drug interactions, and hepatic/renal function. This necessitates long-term therapeutic drug monitoring to optimize treatment efficacy (29, 32).

Due to the side effects of CNIs, there is an increasing clinical demand for alternative drugs, which are currently under investigation. These include corticosteroids, basiliximab, mycophenolate mofetil, and mammalian target of rapamycin (mTOR) inhibitors such as sirolimus and everolimus. However, the immunosuppressive effects of these drugs are relatively weak, and they are typically used in combination with CNIs to balance efficacy and safety. For example, mTOR inhibitors like sirolimus and everolimus have shown significant renal protection after LT (33). The combination of everolimus and low-dose tacrolimus has shown better renal protection than the conventional tacrolimus treatment dose (34). Additionally, mTOR inhibitors have anti-tumor effects, as the phosphoinositide 3-kinase/protein kinase B (Akt)/mTOR signaling pathway plays a crucial role in regulating cell proliferation and apoptosis. Abnormal activation of this pathway is closely associated with tumorigenesis and tumor progression (35). Furthermore, mTOR inhibitors can directly suppress the proliferation of tumor cells, inhibit the growth of endothelial cells, and reduce their response to vascular endothelial growth factor, thereby suppressing angiogenesis and exerting an indirect anti-tumor effect (36). However, the specific treatment regimen for mTOR inhibitors requires exploration through large-scale multicenter clinical trials to obtain better prognostic outcomes.

### 3.3 Personalized immunosuppressive strategies following liver transplantation

Personalized immunosuppressive therapy can effectively reduce rejection episodes and drug toxicity after LT, with biomarker monitoring serving as a cornerstone for treatment optimization: 1) Pharmacogenetic Testing: Tacrolimus pharmacokinetics are significantly influenced by CYP3A5 and CYP3A4 genotypes. Patients expressing CYP3A5 (extensive or intermediate metabolizers) require 1.5–2 times the standard recommended starting dose. The standard recommended starting dose is sufficient for those who do not express CYP3A5 (poor metabolizers) (37). Individuals with the CYP3A4*22 allele have reduced CYP3A4 enzymatic activity, leading to slow tacrolimus metabolism and increased drug concentrations in the blood. In contrast, individuals with the CYP3A4*1B allele may have increased expression of CYP3A4, resulting in faster metabolism of tacrolimus and lower drug concentrations in the blood (38, 39). 2) Immune Cell Function Testing: CD4^+^ T lymphocytes are key to initiating rejection after LT. The cellular immunity function can be reflected by monitoring the adenosine triphosphate (ATP) activity in CD4^+^ T lymphocytes. ImmuKnow (Cylex, Inc., Columbia, MD, USA) uses this principle to measure the activity of CD4^+^ T lymphocytes. In the ImmuKnow test, ATP levels ≤225 ng/mL are interpreted as low immune cell reactivity, while levels ≥525 ng/mL are interpreted as high immune cell reactivity. This information can be used to adjust immunosuppression drug use (40). 3) Cytokine/Chemokine Detection: Cytokines are mediators of immune responses. They have been studied to understand the immune reactions after transplantation. It was shown that the combined detection of IL-10, IL-17, and C-X-C motif chemokine ligand 10 (CXCL10) within two weeks after surgery could predict acute rejection in adult LT recipients, thereby allowing for early intervention (41). Although this article does not provide comprehensive coverage of other associated biomarkers, the integration of multi-omics approaches (encompassing genomic, proteomic, and metabolomic profiling) represents the next frontier in personalized immunosuppressive therapy.

## 4 Conclusion, outlook, and challenges

LT has proven an effective treatment for end-stage liver diseases, significantly improving patient survival and quality of life. The immune microenvironment is critical in regulating graft tolerance and rejection after transplantation. The dynamic balance of immune cells such as T and B cells, particularly the differentiation and functional mechanisms of Th1/Th2 and Th17/Treg cells, provides important insights into the formation of immune tolerance and the development of rejection responses (36). Additionally, exploring new mechanisms such as immune metabolism, DC regulation, and Treg cell-mediated immune tolerance could offer novel directions for post-transplantation immunotherapy strategies. However, current immunosuppressive strategies primarily rely on CNIs such as cyclosporine and tacrolimus. Achieving a balance between their therapeutic efficacy and toxicity remains a major challenge (33). Post-LT immunosuppressive therapy faces multiple significant challenges. The side effects caused by excessive immunosuppression severely impact patients’ quality of life and long-term survival. Conversely, insufficient immunosuppression might trigger rejection responses, leading to graft dysfunction and structural degradation. Additionally, the clinical application of new technologies faces issues such as high costs, poor predictability of efficacy, and unknown long-term safety.

In-depth research into the immune microenvironment and tolerance regulation mechanisms of LT is essential to achieving better outcomes. Exploring the interactions among cells and related cytokines in the immune microenvironment regulation network will aid in developing more targeted and less toxic immunosuppressants or biologics, optimizing current immunotherapy strategies. Particularly, integrating emerging molecular imaging technologies to construct imaging probes targeting key biomarkers of the immune microenvironment could enable dynamic monitoring of post-transplantation immune responses and therapy efficacy (42). This approach holds promise in improving the current challenges in post-transplantation immunotherapy.
